# Early retinal functional alteration in relation to diabetes duration in patients with type 2 diabetes without diabetic retinopathy

**DOI:** 10.1038/s41598-022-15425-x

**Published:** 2022-07-06

**Authors:** Sangeetha Srinivasan, Sobha Sivaprasad, Ramachandran Rajalakshmi, Ranjit Mohan Anjana, Rayaz A. Malik, Vaitheeswaran Kulothungan, Viswanathan Natarajan, Rajiv Raman, Muna Bhende

**Affiliations:** 1 Vision Research Foundation, Chennai, India; 2 NIHR Moorfields Biomedical Research Centre, London, UK; 3 Dr. Mohan’s Diabetes Specialties Centre and Madras Diabetes Research Foundation, Chennai, India; 4 Weill Cornell Medicine-Qatar, Education City, Doha, Qatar; 5 Central Manchester University Hospitals Foundation Trust, Manchester, UK; 6 National Centre for Disease Informatics and Research (NCDIR) & Indian Council of Medical Research (ICMR), Bangalore, India; 7 Department of Preventive Ophthalmology, Sankara Nethralaya, Chennai, India; 8 Shri Bhagwan Mahavir Vitreoretinal Services, Sankara Nethralaya, Chennai, India

**Keywords:** Outcomes research, Diseases

## Abstract

To examine the retinal structure and function in relation to diabetes duration and glycemia in patients without diabetic retinopathy (DR). 85 adults with type 2 diabetes without DR or macular edema underwent dilated indirect ophthalmoscopy, optical coherence tomography (OCT), ultra-wide field fundus photography, multifocal electroretinography (mfERG) and HbA_1C_ assessment. Patients were stratified as those with diabetes duration < 10 years and ≥ 10 years. Right eyes of all participants were analyzed. mfERG was analysed as ring 12, 34, 56. No significant differences were noted in OCT-derived retinal thickness measures between groups. mfERG P1 latencies were delayed, and amplitudes (nV/deg^2^) were reduced in all three rings in those with diabetes duration ≥ 10 years vs. < 10 years, with significant correlations to diabetes duration in all rings. Logistic regression showed that duration of diabetes ≥ 10 years was associated with greater age (odds ratio (OR) 1.081, 95% CI 1.022, 1.143) and lower P1 amplitudes in the middle ring (OR 0.924, 95% CI 0.854, 0.999). No significant correlations were observed between HbA_1c_ and retinal measures. In the absence of DR, early retinal functional alterations are detectable on mfERG in patients with longer diabetes duration, but with no difference in OCT-derived retinal thickness.

## Introduction

About 1 in 10 people aged 20–79 years world-wide have diabetes^1^. A recent global systematic review and meta-analysis established that global prevalence was 22.27% (95% confidence interval [CI] 19.73–25.03%) for diabetic retinopathy (DR), 6.17% (95% CI 5.43–6.98%) for vision threatening DR, and 4.07% (95% CI 3.42–4.82%) for clinically significant macular edema, and disproportionately affected countries in the Middle East, North Africa and the Western Pacific^2^.

Vascular lesions that define DR are visible manifestations of damage to the retinal microvasculature^3^ and have largely been attributed to prolonged duration of diabetes^4^ and poor glycemic control^5,6^. However, diabetic retinal disease is now increasingly recognized to affect both the retinal microvasculature and neurons. Over time, these changes may result in impairment of visual function such as reduced contrast sensitivity, impaired colour vision and defects in the visual field^7–10^.

These changes have been related to the presence and severity of DR^11–13^. However, recent methods to assess early structural and functional alterations in the retina such as optical coherence tomography (OCT), OCT-angiography (OCT-A) and multifocal electroretinography (mfERG)^14^ have shown abnormalities in patients without DR^15–17^ compared to healthy individuals. The focus of this study was to examine the retinal structure and function in relation to diabetes duration and HbA_1c_ in individuals with diabetes without clinical signs of DR.

The current study assessed the relationship of retinal parameters with the duration of diabetes and HbA_1c_ levels in individuals with type 2 diabetes without DR. OCT-derived retinal thickness measures and retinal function with mfERG were examined. Outcome measures were full retinal thickness measures, retinal nerve fibre layer (RNFL) and ganglion cell layer-inner plexiform layer thicknesses (GCL + IPL), mfERG P1 latencies and amplitudes in three rings, contrast sensitivity and visual acuity.

## Participants and methods

This was a prospective study conducted at a tertiary eye hospital in Chennai, South India, approved by the Institutional Review Board, Vision Research Foundation, Chennai, India (Ethics approval no: 642-2017-P). The study followed the tenets of the Declaration of Helsinki. We present the baseline data from a 4-year longitudinal study in participants recruited between March 2018 to July 2019. Participants provided written informed consent.

Participants were consecutively recruited from the vitreoretinal outpatient department of the hospital. Individuals with type 2 diabetes of at least 1-year duration were screened Exclusion criteria were individuals who could not give informed consent, inability to maintain fixation on mfERG or if they indicated that they were unable to attend annual follow-up visits. Individuals with media haziness that compromised visual acuity or quality of imaging, coexisting ocular infection or inflammation, spherical refractive error greater than ± 6D, astigmatism greater than ± 3D, IOP > 22 mmHg, a vertical and horizontal cup-disc ratio > 0.6 or reasonable suspicion of glaucoma from optic nerve head appearance, those who had undergone or planned for vitreoretinal surgery, retinal vascular occlusion, and those participating in any interventional studies were excluded. Those with any stage of DR, with or without diabetic macular edema (DME) were excluded. DME was considered to be present if there was evidence of retinal thickening or hard exudates in the posterior pole, as observed on dilated indirect ophthalmoscopy. DME was also defined based on OCT as a foveal thickness > 300 μm^18^.

Individuals underwent visual acuity testing, objective and subjective refraction, intraocular pressure assessment, cataract grading after pupillary dilatation and ultra-wide field fundus photography (Optos UWF™, Optos Inc, UK), HbA_1c_ and systolic and diastolic blood pressure assessment. Best corrected visual acuity (BCVA) was recorded on Snellen’s chart and converted to LogMAR acuity. Participants were required to have a minimum BCVA of 20/80 (6/24) for fixation on mfERG. Cataract grading was undertaken after pupillary dilatation by one observer (SS) using a slit-lamp (SL-120; Carl Zeiss Meditec, Jena, Germany) per LOCS III standard photographs (LOCS III; LOCS chart III; Leo T Chylack, Harvard Medical School, Boston, MA, USA).

### Optical coherence tomography

The Cirrus HD-OCT 5000, Carl Zeiss meditec, USA was utilized for the assessment of retinal thickness measures. Macular thickness was assessed from the internal limiting membrane to the retinal pigment epithelium using the macular cube 512 × 128 protocol in the nine zones; the optic disc cube 200 × 200 scan was utilized for the retinal nerve fibre layer thickness (RNFL) assessment. The ganglion cell layer+inner plexiform layer thickness (GCL + IPL) was generated from the macula cube protocol. OCT scans with signal strength < 6 were excluded from the study.

### Multifocal electroretinography

**Figure 1:**
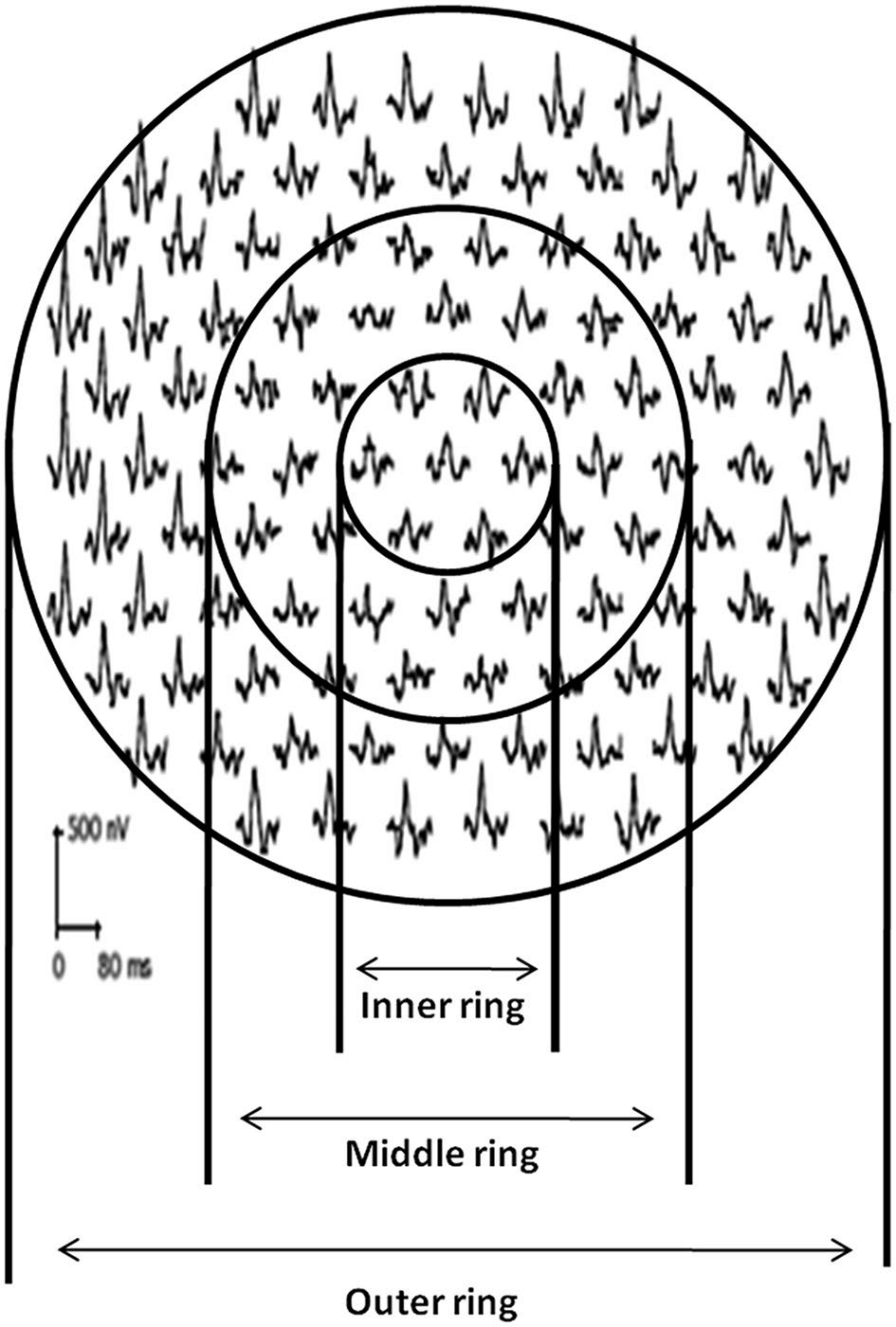
Grouping of mfERG rings as inner ring (rings 12), middle ring (ring 34) and outer ring (ring 56) as described by Seiple et al.^18^.

Participants underwent multifocal electroretinogram (mfERG) (Veris™ Science 6.4.8 app, California, USA) assessment based on the International Society for Clinical Electrophysiology of Vision (ISCEV) guidelines. Testing was done using a Burian Allen Electrode, uniocularly with refractive correction in place, and the other eye was patched. A gold cup electrode attached to the earlobe served as a ground electrode. The stimulus for mfERG consisted of an array of 103 hexagons presented on a monitor at a frame rate of 75 Hz, subtending an angle of 35 degrees horizontally and 31 degrees vertically at a viewing distance of 53 cm, flickering according to a pseudorandom m-sequence at a mean luminance of 64 cd/m^2^. The luminance of the bright and the dark hexagons were 128 cd/m^2^ and 1 cd/m^2^, respectively. For fixation, a red cross of 2 mm diameter was used, and an in-built camera enabled the operator to monitor fixation throughout the recording. An internal Grass amplifier (Grass Technologies, An Astro-Med, Inc, West Warwick, R.I.) amplified the recordings (6100 000) which were then band-pass filtered (10–100 Hz). The actual mfERG recording time was 7 min and 17 s per eye. The mfERG P1 responses (mathematical extractions) were analyzed using the Veris software and the first-order kernels were recorded and displayed in the form of a trace array of 103 local retinal responses, a 3-dimensional topographical chart. The P1 amplitude was obtained from the first negative trough to the first positive peak and the P1 latency was assessed from stimulus onset to first positive peak. In order to maintain a steady fixation on the fixation target in mfERG, a minimum acuity of 20/80 (6/24) was considered a pre-requisite. The mfERG rings were then examined as 3 rings as described by Seiple et al.^19^. P1 measures in rings 1 and 2 were averaged and referred to as ‘ring 12’; rings 3 and 4 were averaged and referred to as ‘ring 23’ and rings 5 and 6 were averaged and referred to as ‘ring 56’ (Fig. 1).

The Pelli-Robson contrast sensitivity chart (Metropia Ltd, Cambridge, UK) was used to assess the contrast sensitivity (CS), at a recommended testing distance of 1 m. The chart has six letters in each row arranged in groups of three, each group having an individual contrast. Participants were asked to read the letters from left to right and then the next line, starting with the highest contrast, until they are unable to read at least two of the three letters in a group. A numerical score was then assigned based on a minimum of 2 letters out of a triplet with the same photometric contrast and was recorded as the lowest contrast correctly read (indication of having reached threshold) and expressed in log units.

### Statistical analysis

All tests were performed in both eyes of the participants. However, for statistical analysis, only measures in the right eyes of participants were analyzed. Participants were grouped according to duration of diabetes ≥ 10 years and < 10 years, and HbA1c ≥ 7.0 and HbA1c < 7%. The cut-off of diabetes duration of ≥ 10 years versus < 10 years was chosen so that the patient numbers were comparable and roughly equally distributed in both groups. Continuous variables were assessed for normality of distribution. A student t-test was used to compare between groups for normally distributed data and a Mann–Whitney U test was used for non-normally distributed data. Univariate and multivariable binary logistic regression was performed with the duration of diabetes as the outcome variable. A p-value < 0.05 on group comparison was considered statistically significant and the relationship between variables was assessed in univariate regression. Those with a p-value < 0.05 on univariate regression were entered into multivariable binary logistic regression analysis to examine for factors associated with diabetes duration.

## Results

### Baseline clinical characteristics and diabetes duration

Eighty-five individuals fit the eligibility criteria. Table 1 shows the baseline clinical characteristics in those with diabetes duration ≥ 10 years and < 10 years. The age range in the entire group was 37–82 years, with a mean of 59.5 ± 8.7 years. The mean age in those with diabetes duration ≥ 10 years was significantly higher compared to those with diabetes duration < 10 years (56.3 ± 8.7 years vs. 62.5 ± 7.6, p < 0.001). The mean duration of diabetes in those with duration < 10 years was 5.2 ± 1.9 years and 15.2 ± 4.9 years in those with duration ≥ 10 years (p < 0.001). Gender distribution (p = 0.231), use of insulin (p = 0.809) history of hypertension (p = 0.944), history of heart disease (p = 1.00) systolic (p = 0.502) and diastolic blood pressures (p = 0.053), HbA_1c_ levels (p = 0.380) and lipid profiles did not differ between the two groups. The spot urine albumin (Microalbumin) levels were higher in the group with diabetes duration ≥ 10 years compared to < 10 years (83.87 ± 257.20 vs 28.99 ± 52.65 mg/dL, p = 0.004).

**Table 1:** Clinical, optical, OCT and mfERG measures stratified according to diabetes duration.

Diabetes, No DR	Diabetes duration < 10 years n = 43		Diabetes duration ≥ 10 years n = 42		
	Mean	SD	Mean	SD	p-values
**Clinical and ophthalmic measures**					
Age, years	56.3	8.7	62.5	7.6	< 0.001
Men, n (%)		22 (51.1%)		27 (64%)	0.231
Diabetes duration, years	5.2	1.9	15.2	4.9	< 0.001
HbA_1c_, %	8.7	2.7	8.1	1.8	0.380
Use of insulin (yes), n (%)	8 (18.6%)		7 (16.6%)		0.809
Hypertension (yes), n (%)	30 (69.7%)		29 (69.0%)		0.944
Heart disease (yes), n (%)	1 (2.3%)		1 (2.3%)		1.00
Total cholesterol, mg/dL	196.74	49.90	190.20	44.38	0.638
LDL, mg/dL	114.94	36.00	108.96	35.26	0.439
Triacyl glycerol, mg/dL	195.82	108.02	183.49	74.57	0.984
VLDL, mg/dL	35.77	15.22	35.71	13.45	0.838
Spot urine albumin (microalbumin), mg/dL	28.99	52.65	83.87	257.20	0.004
Sys BP, mmHg	132	15	136	18.36	0.502
Dia BP, mmHg	73	8	77	9.21	0.053
Sp Eq, dioptre	− 0.07	1.84	0.53	2.04	0.231
LogMAR	0.04	0.13	0.04	0.1	0.425
CS, log units	1.30	0.20	1.31	0.18	0.987
Phakic, n (%)		34 (79%)	30	(71.4%)	0.414
Pseudophakic, n (%)		9 (21%)	12	(28.6%)	
Bonferroni’s adjusted p-value for demographics, clinical variables and ophthalmic variables is p = 0.002					
**OCT measures, µm**					
Fovea	239	20	238	21	0.671
Inner superior	311	24	312	19	0.754
Inner inferior	309	22	311	22	0.792
Inner temporal	298	22	303	26	0.998
Inner nasal	310	23	314	23	0.819
Outer superior	271	16	273	16	0.727
Outer inferior	263	16	261	14	0.224
Outer temporal	255	15	255	27	0.671
Outer nasal	293	22	293	17	0.665
Cube avg. thickness	273	16	273	15	0.522
RNFL	90.46	11.98	91.36	8.55	0.783
GCL + IPL	79.95	8.89	79.28	6.76	0.174
Bonferroni’s adjusted p-value for OCT measures is p = 0.004					
**mfERG P1 measures**					
meanP1 amp ring 12	27.296	11.433	22.951	7.526	0.033
meanP1 amp ring 34	17.596	6.882	13.909	5.296	0.007
meanP1 amp ring 56	13.794	5.887	10.817	4.590	0.014
meanP1 lat ring 12	30.871	2.583	31.780	2.044	0.009
meanP1 lat ring 34	29.640	1.863	30.283	1.695	0.006
meanP1 lat ring 56	29.859	1.509	30.551	1.593	0.003
Bonferroni’s adjusted p-value is p = 0.008 for mfERG measures					

*DR* diabetic retinopathy, *BP* blood pressure, *Sp Eq* spherical equivalent, *LogMAR* Logarithm of the minimum angle of resolution, *CS* contrast sensitivity, *RNFL* retinal nerve fibre layer, *GCL + IPL* ganglion cell layer + inner plexiform layer, *Lat* Latencies expressed as milliseconds, *amp* amplitude assessed as response density, nV/deg^2^.

The refractive error assessed as spherical equivalent (p = 0.231), visual acuity (by Logarithm of the Minimum Angle of Resolution, LogMAR) (p = 0.425), contrast sensitivity (p = 0.987) and phakic status (p = 0.414) did not differ significantly between the two groups.

### OCT-derived retinal thickness measures and diabetes duration

Table 1 provides a summary of the OCT-derived macular thickness, RNFL and GCL + IPL thickness measures according to diabetes duration. There were no significant differences between the two groups for OCT-derived retinal parameters. In those with diabetes duration < 10 years, the minimum to maximum foveal thickness was 182 µm to 280 µm, and 195 µm to 274 µm in those with diabetes duration ≥ 10 years. Therefore, none of the patients had DME based on OCT definition.

### Retinal functional measures and diabetes duration

Retinal functional measures assessed by mfERG in the three rings with respect to diabetes duration are summarized in Table 1. The P1 response densities in all three rings were reduced and the latencies in all three rings were delayed in those with diabetes duration ≥ 10 years compared to diabetes duration < 10 years. After Bonferroni’s correction for multiple comparisons (p = 0.008), the P1 amplitudes in ring 34 and latencies in rings 34 and 56 remained statistically significant.

There were no significant differences in mfERG measures between phakics and pseudophakics. In addition, there was no significant relationship between lens status and duration of diabetes (chi-square = 0.667, p = 0.414) (Supplementary Table S1).

### OCT-–derived retinal thickness measures and retinal functional measures and HbA_1c_

OCT-derived retinal thickness measures and the mfERG measures did not differ between participants with HbA1c < 7% compared to HbA_1c_ ≥ 7.0 (Table 2). OCT-derived retinal thickness measures did not differ for HbA_1c_ cut-offs of ≥ 6.0%, ≥ 6.5% and ≥ 7.5%.

**Table 2:** OCT and mfERG measures stratified according to HbA_1c_.

OCT thickness measures	HbA_1c_ < 7, n = 28		HbA_1c_ ≥ 7.0, n = 57		p-values
	Mean	SD	Mean	SD	
Fovea	237.93	18.403	239.16	21.115	0.848
Inner superior	311.54	17.698	312.68	23.181	0.653
Inner inferior	307.75	19.946	311.33	22.543	0.531
Inner temporal	299.39	16.024	301.84	26.265	0.64
Inner nasal	312.96	18.761	311.61	24.889	0.786
Outer superior	270.36	12.734	274.63	21.583	0.674
Outer inferior	259.82	13.768	263.47	14.732	0.379
Outer temporal	255.54	13.254	256.75	25.057	0.885
Outer nasal	289.64	19.097	293.71	20.706	0.439
Cube avg. thickness	271.25	13.25	274.42	16.22	0.51
Average RNFL	92.85	9.51	90.94	9.443	0.292
Average GCL + IPL	79.81	6.52	79.87	8.01	0.94
**mfERG measures**					
meanP1 amp ring 12	25.711	9.761	25.052	10.473	0.702
meanP1 amp ring 34	16.253	6.496	15.249	6.455	0.371
meanP1 amp ring 56	12.361	5.511	11.916	5.259	0.571
meanP1 lat ring 12	31.092	2.361	31.761	2.683	0.183
meanP1 lat ring 34	30.024	1.944	30.170	2.126	0.436
meanP1 lat ring 56	30.154	1.737	30.404	1.711	0.328

Latencies expressed as milliseconds; amplitude assessed as response density, nV/deg^2^.

### Univariate correlations and multivariate logistic regression

Univariate correlations were assessed between mfERG measures, diabetes duration and HbA_1c_ levels. mfERG measures showed significant correlations with diabetes duration, but not with HbA_1c_ (Table 3). Univariate correlations were also assessed between microalbuminuria and mfERG P1 measures in the two duration groups and were not significant.

**Table 3:** Correlation between mfERG measures with HbA_1c_ and diabetes duration.

Correlation between mfERG, duration, HbA_1c_	Diabetes duration		HbA_1c_	
	Correlation coefficient	p-values	Correlation coefficient	p-values
meanP1 amp ring 12	− 0.247	0.021	− 0.0001	0.998
meanP1 amp ring 34	− 0.300	0.004	− 0.046	0.673
meanP1 amp ring 56	− 0.284	0.007	− 0.001	0.994
meanP1 lat ring 12	0.226	0.035	0.194	0.072
meanP1 lat ring 34	0.299	0.004	0.140	0.197
meanP1 lat ring 56	0.343	0.001	0.132	0.227

*Lat* Latencies expressed as milliseconds; *amp* amplitude assessed as response density, nV/deg^2^.

Table 4 shows univariate and multivariable regression analysis. On univariate regression analysis, greater age (p < 0.001) and P1 amplitudes of ring 34 (p = 0.004) and 56 (p = 0.020) were significantly related to duration of diabetes ≥ 10 years. Since amplitudes in the three rings may be correlated to each other, they were entered in separate multivariable models. On entering age and middle ring amplitude (ring 34) into multivariable analysis (model 1), greater age (OR 1.081, 95% CI 1.022, 1.143) and amplitude in ring 34 (OR 0.924, 95% CI 0.854, 0.999) were significantly and independently associated with diabetes duration ≥ 10 years. On entering age and outer ring amplitude (ring 56) into multivariable analysis (model 2), only age was significantly associated with diabetes duration ≥ 10 years (OR 1.087, 95% CI 1.028, 1.149).

**Table 4:** Association between duration of diabetes, age and mfERG measures per mfERG rings.

Regression: amplitudes with duration ≥ 10 years						
	B	S.E	p-values	OR	95% CI for OR	
					Lower	Upper
**mfERG P1 amplitudes vs. duration ≥10 years**						
**Univariate regression**						
Age	0.095	0.028	**< 0.001**	1.100	1.042	1.161
meanP1 amp ring 12 (Inner ring)	− 0.045	0.023	0.051	0.956	0.913	1.000
meanP1 amp ring 34 (Middle ring)	− 0.108	0.038	**0.004**	0.898	0.833	0.967
meanP1 amp ring 56 (Outer ring)	− 0.095	0.041	**0.020**	0.909	0.840	0.985
**Multivariable regression**						
Model 1 with middle ring amplitude						
Age	0.078	0.029	**0.006**	1.081	1.022	1.143
meanP1 amp ring 34 (Middle ring)	− 0.079	0.040	**0.047**	0.924	0.854	0.999
Model 2 with outer ring amplitude						
Age	0.083	0.028	**0.003**	1.087	1.028	1.149
meanP1 amp 56 (Outer ring)	− 0.065	0.043	0.124	0.937	0.862	1.018

Regression: latencies with duration ≥ 10 years						
	B	S.E	p-values	OR	Lower	Upper
**mfERG P1 latencies vs. duration ≥ 10 years**						
**Univariate regression**						
meanP1 lat ring 12 (Inner ring)	0.117	0.085	0.171	1.124	0.951	1.328
meanP1 lat ring 34 (Middle ring)	0.213	0.127	0.096	1.237	0.963	1.588
meanP1 lat ring 56 (Outer ring)	0.329	0.153	**0.031**	1.390	1.031	1.874
**Multivariable regression**						
Age	0.081	0.029	**0.005**	1.084	1.024	1.147
meanP1 lat ring 56 (Outer ring)	0.204	0.165	0.215	1.226	0.888	1.694

Significant values are in bold.

Variables entered into equation are chosen whose p-values were < 0.05 on univariate analysis; *lat* latencies, *amp* amplitudes (nV/deg^2^).

On univariate regression, the P1 latencies in ring 56 showed a significant relation to diabetes duration ≥ 10 years (p = 0.031). On multivariable regression along with age, only age showed a significant association with diabetes duration ≥ 10 years (OR 1.084, 95% CI 1.024, 1.147).

## Discussion

We examined for retinal structural and functional alterations in those with diabetes duration ≥ 10 years in comparison to those with diabetes duration < 10 years. The primary findings of the study are that the mfERG measures are altered in individuals with diabetes in relation to longer diabetes duration. These findings are observed in the absence of significant alterations to the macular thickness, RNFL, GCL + IPL thicknesses or clinical signs of DR. Previous studies^20–23^ have demonstrated a reduction in P1 amplitudes and a delay in latencies in subjects with type 2 diabetes without DR compared to healthy controls, with a negative correlation between P1 amplitude and diabetes duration, and a positive correlation between P1 latency and diabetes duration^22^. It is likely that the retinal functional alterations may start in people with prediabetes^24^.

In the current study, the amplitudes and latencies gradually decreased from the inner to outer ring in both the groups. (Table 1) More specifically, in those with diabetes duration ≥ 10 years, the mfERG amplitude in ring 34 (middle ring) was lower when compared to the other rings (Table 4) Balta et al.^20^ demonstrated reduced P1 amplitudes in the inner retinal areas (ring 1 and ring 2) and a delayed P1 latency only in ring 2 in individuals with diabetes without retinopathy, with a correlation between diabetes duration and P1 amplitudes. Furthermore, Adhikari et al.^25^ observed significant correlations between P1 amplitudes and latencies only in rings 3–6, indicating regional differences in retinal function. Similar regional differences have been reported in healthy controls. Ghatak et al.^26^ showed that P1 amplitudes decrease, and latencies increase from the central to peripheral retina in normal emmetropic subjects. Mohidin et al.^27^ showed no significant differences in overall amplitude density in 90 healthy subjects aged 18–52 years stratified into three age groups, but there were significant differences between rings at different eccentricities. In our study, although the upper limit for the odds ratio for mean P1 amp ring 34 was 0.999, it still indicates early retinal functional changes in relation to diabetes duration ≥ 10 years.

The decline in retinal function in diabetes may be attributed to age^28^, neural factors^27–29^ or both^30^. Seiple et al.^19^ studied 62 healthy individuals aged 21–81 years and showed that the P1 amplitude decreased by 10.5%, while latency increased by 1.0%, per decade increase in age. Tzekov et al.^31^ in healthy subjects reported a 5% decline in P1 amplitudes per decade increase in age in subjects aged 9–80 years. In our study, a 15% decrease was noted in those with duration ≥ 10 years for a 6-year difference in the mean age between the two groups (Table 1) indicating that diabetes duration has an additive effect. In the current study, mfERG was performed after dilatation; there were no significant differences in spherical equivalent or LogMAR visual acuity between the two groups. OCT images with signal strength < 6.0 were excluded. In addition, no significant differences were observed in mfERG measures between phakic and pseudophakic eyes, indicating that optical factors may have less likely influenced our results.

We observed no significant correlation between HbA_1c_ and retinal measures. Adhikari et al.^25^ also observed no significant correlation between P1 amplitude and latency with fasting blood glucose levels. Kim et al.^32^ demonstrated no effect of glycemia on mfERG measures. A lack of association between mfERG measures and HbA_1c_ has also been reported in the European Consortium for the Early Treatment of Diabetic Retinopathy (EUROCONDOR) study^8^. One explanation could be that whilst HbA_1c_ reflects a three-month average of glycemia, a longer-term assessment of glycemia is required with serial HbA_1c_ measurement. Indeed, OCT-derived retinal thicknesses and mfERG measures with HbA_1c_cut-offs of ≥ 6.0%, ≥ 6.5% and ≥ 7.5%, did not differ significantly between groups. Retinal functional measures may vary with variations in HbA_1c_, but serial HbA_1c_ was not available to assess for HbA_1c_ variability in the current study.

In the current study, 18.6% of patients with diabetes duration < 10 years and 16.6% of patients with diabetes duration ≥ 10 years were on insulin in addition to other antidiabetic drugs. Glucagon-Like Peptide (GLP)-1 drugs have shown beneficial effects in experimental diabetic retinopathy (DR) through their neuroprotective and antioxidant properties^33^; however, none of the patients in the current study were on GLP-1 agonists. We observed altered mfERG measures in individuals with diabetes in relation to longer duration of diabetes in the absence of retinal structural alterations on OCT. Subclinical retinal functional alterations in diabetes prior to visible signs of DR has been previously reported in animal models of hyperglycemia^34^ as well as in humans^16^.

These alterations have been linked to complex mechanisms such as increased oxidative stress, inflammation, loss of neuroprotective factors and glutamate excitotoxicity in long-standing diabetes^35–39^. Hyperglycemia is reported to disrupt the metabolic environment in the retina, which affects neuronal survival, leading to early neuronal cell death^36^. In our study, HbA_1c_ levels were comparable in the two groups. However, the mfERG responses (a measure of retinal function) showed alterations in relation to longer duration of diabetes in the absence of retinal structural changes on OCT. This indicates neuroretinal dysfunction rather than degeneration. More specifically, the changes underlying the observed delays in mfERG response and reduction in amplitudes may indicate retinal hypoxia^40,41^ associated with early or undetected perfusion defects from dysfunction of retinal capillaries^35,36,42,43^.

Studies in animal models demonstrated that vascular permeability, longevity of the retinal cells and normal functioning of the retina is maintained by glial cells, namely Müller cells and astrocytes. Studies report gliosis, changes in Müller cell neurotransmitter and ion channel functioning, alterations to growth factors in diabetes, and decreased astrocyte communication have been linked to neuronal dysfunction^44,45^.

Since the Müller cells process extends to the photoreceptor inner segments, a dysfunction of glial cells also affects the photoreceptors and some of the inner retinal layers early in diabetes. As a result, there is disruption to the normal functioning of multiple retinal layers involving photoreceptors, glial cells and ON and OFF bipolar cells in diabetes^46–48^. Animal studies demonstrate that in the early weeks of inducing diabetes, inner retinal dysfunction involving amacrine and ganglion cells is evident, followed by outer retinal compromise later^49,50^. Clinically, these changes are reported to be precursors to impending visible vascular changes in the retina.

Our study has certain limitations. The duration of diabetes was self-reported and can of course vary by 5–10 years from the actual onset. We did not evaluate the impact of diabetic neuropathy. However, we did observe greater albuminuria in patients with a longer duration of diabetes, which is consistent with the thesis that microvascular complications go hand in hand. Detailed examination of the retinal structure and visual and retinal function in a cohort of individuals with T2DM without DR based on ultra-wide field fundus photography has generated novel data on early retinal dysfunction whilst taking into account important confounding factors.

In conclusion, patients with type 2 diabetes without DR have evidence of early retinal dysfunction detectable by mfERG in the absence of any significant abnormalities in the retinal structure examined using ultra-wide field retinal fundus photography and OCT imaging.

## Supplementary Information

Supplementary Information.

## Data Availability

Data are available from the corresponding author upon request.
