# Supplementary material for: Early retinal functional alteration in relation to diabetes duration in patients with type 2 diabetes without diabetic retinopathy
Source: Sci Rep. 2022 Jul 6;12:11422. doi: 10.1038/s41598-022-15425-x (PMC9259684; doi:10.1038/s41598-022-15425-x)
Supplement: Supplementary file 1 — Supplementary Information. [file 41598_2022_15425_MOESM1_ESM.docx]

**Early retinal functional alteration in relation to diabetes duration in patients with Type 2 diabetes without diabetic retinopathy**

Sangeetha Srinivasan, PhD,^1^ Sobha Sivaprasad, DM,^2^ Ramachandran Rajalakshmi, DO,^3^Ranjit Mohan Anjana, MD,^3^Rayaz A. Malik, MBChB,^4^ Vaitheeswaran Kulothungan, PhD,^5^ Viswanathan Natarajan, MSc,^6^ Rajiv Raman, Hon.DSc,^7^ Muna Bhende,MS.^7^

Table S1. mfERG measures in phakic versus pseudophakic eyes

|  | Phakic n=64 | | Pseudophakic n=21 | | M-W |
| --- | --- | --- | --- | --- | --- |
| mfERG measures | Mean | SD | Mean | SD | p-values |
| mean of amplitudes 1 and 2 rings, nV/deg^2^ | 25.704 | 10.709 | 24.152 | 8.448 | 0.624 |
| mean of amplitudes 3 and 4rings, nV/deg^2^ | 15.972 | 6.513 | 14.878 | 6.534 | 0.575 |
| mean of amplitudes 5 and 6 rings, nV/deg^2^ | 12.206 | 5.268 | 12.127 | 6.193 | 0.697 |
| mean of latencies 1 and 2 rings, ms | 31.452 | 2.669 | 31.465 | 2.460 | 0.996 |
| mean of latencies 3 and 4rings, ms | 30.054 | 2.058 | 30.102 | 2.086 | 0.658 |
| mean of latencies 5 and 6 rings, ms | 30.257 | 1.621 | 30.327 | 2.022 | 0.457 |

SD, standard deviation; M-W, Mann-Whitney U test; Amplitudes assessed as response densities in nV/deg^2^
